# The use of sodium DL-3-Hydroxybutyrate in severe acute neuro-metabolic compromise in patients with inherited ketone body synthetic disorders

**DOI:** 10.1186/s13023-020-1316-x

**Published:** 2020-02-18

**Authors:** Kaustuv Bhattacharya, Walid Matar, Adviye Ayper Tolun, Beena Devanapalli, Sue Thompson, Troy Dalkeith, Kate Lichkus, Michel Tchan

**Affiliations:** 1 Disciplines of Genetic Medicine and Child and Adolescent Health, University of Sydney, Sydney, Australia; 2 Genetic Metabolic Disorders Service, Sydney Children’s Hospital Network, Children’s Hospital at Westmead, Locked Bag 4001, Westmead, NSW 2145 Australia; 3 Department of Neurology, St George Hospital, Kogarah, NSW Australia; 4 NSW Biochemical Genetics Service, Westmead, NSW Australia; 5 Westmead Hospital, University of Sydney, Westmead, Australia

**Keywords:** Carnitine acyl-carnitine translocase deficiency (CACTD), 3-hydroxyl-3-methylglutaryl-CoA lyase deficiency (HMGCLD), Carnitine palmitoyl transferase II deficiency (CPT2D), Ketone body, 3-hydroxybutyrate, Fat oxidation

## Abstract

**Background:**

Ketone bodies form a vital energy source for end organs in a variety of physiological circumstances. At different times, the heart, brain and skeletal muscle in particular can use ketones as a primary substrate. Failure to generate ketones in such circumstances leads to compromised energy delivery, critical end-organ dysfunction and potentially death. There are a range of inborn errors of metabolism (IEM) affecting ketone body production that can present in this way, including disorders of carnitine transport into the mitochondrion, mitochondrial fatty acid oxidation deficiencies (MFAOD) and ketone body synthesis. In situations of acute energy deficit, management of IEM typically entails circumventing the enzyme deficiency with replenishment of energy requirements. Due to profound multi-organ failure it is often difficult to provide optimal enteral therapy in such situations and rescue with sodium DL-3-hydroxybutyrate (S DL-3-OHB) has been attempted in these conditions as documented in this paper.

**Results:**

We present 3 cases of metabolic decompensation, one with carnitine-acyl-carnitine translocase deficiency (CACTD) another with 3-hydroxyl, 3-methyl, glutaryl CoA lyase deficiency (HMGCLD) and a third with carnitine palmitoyl transferase II deficiency (CPT2D). All of these disorders are frequently associated with death in circumstance where catastrophic acute metabolic deterioration occurs. Intensive therapy with adjunctive S DL-3OHB led to rapid and sustained recovery in all. Alternative therapies are scarce in these situations.

**Conclusion:**

S DL-3-OHB has been utilised in multiple acyl co A dehydrogenase deficiency (MADD) in cases with acute neurological and cardiac compromise with long-term data awaiting publication. The use of S DL-3-OHB is novel in non-MADD fat oxidation disorders and contribute to the argument for more widespread use.

## Introduction

Mitochondrial fatty acid oxidation (MFAO) disorders comprise disorders that lead to impaired production of the ketone bodies in the liver. MFAO yields acetyl-Co A which condenses to form the ketone body acetoacetate [1]. The other ketone bodies (KB), 3-hydroxybutyrate and acetone are derived from acetoacetate. Failure to generate KB at times of physiological stress has catastrophic sequelae in MFAO and KB synthetic disorders [2]. In severe forms of these conditions, such as CACTD, HMGCL2 deficiency and CPT2D, life threatening encephalopathy, cardiomyopathy and arrythmias during metabolic decompensation are well recognised [3–8]. Chronic treatment generally utilises medium chain triglyceride (MCT) as an alternative energy source, but this would be ineffective in HMGCL2 deficiency [6, 9]. In acute metabolic decompensation, intravenous carbohydrate is used though adequate caloric supplementation sufficient to reverse catabolism is difficult to achieve. The three cases presented in this paper with CACTD, HMGLC2D and CPT2D were all cases in which catastrophic acute encephalopathy was progressing despite management with intravenous dextrose saline. Intervention with S DL-3-OHB reversed this course and led to sustained recovery in all. S DL-3-OHB has previously been used in MADD in similar critical situations leading to recovery but not in other fat oxidation disorders [10].

## Methods

The use of S DL-3-OHBwas granted by our institution’s therapeutic drug and ethics committee. In all cases notification of emergency administration and ongoing supply was made via the Special Access Scheme of the Australian Federal Government’s Therapeutic Goods Administration (TGA.) The TGA serves as a similar regulatory body to the Food and Drugs Administration (FDA) in USA. This study has been approved by the Ethics Committee of Sydney Children’s Hospitals’ Network and informed consent was taken from the patient or carer as appropriate for age. The report comprises retrospective chart review.

Urinary organic acid levels were analysed qualitatively using gas chromatography mass spectrometry (GC/MS) (QP-2010 Ultra, Shimadzu Corp., Japan) after solvent extraction and trimethylsilyl derivatization of urine samples as previously described [11, 12]. Underivatized plasma acylcarnitine levels were analysed using ultra-high performance liquid chromatography-tandem mass spectrometry (UPLC-MS/MS) (Xevo TQ-S Acquity UPLC, Waters, USA) [13, 14]. CPT II enzyme analysis on leucocytes and fibroblasts were based on a modified version of Demaugre et al., 1991 using non-radioactive substrates. Protein content was determined using bicinchoninic assay (BCA) [15, 16].

### Case I

**Fig. 1:**
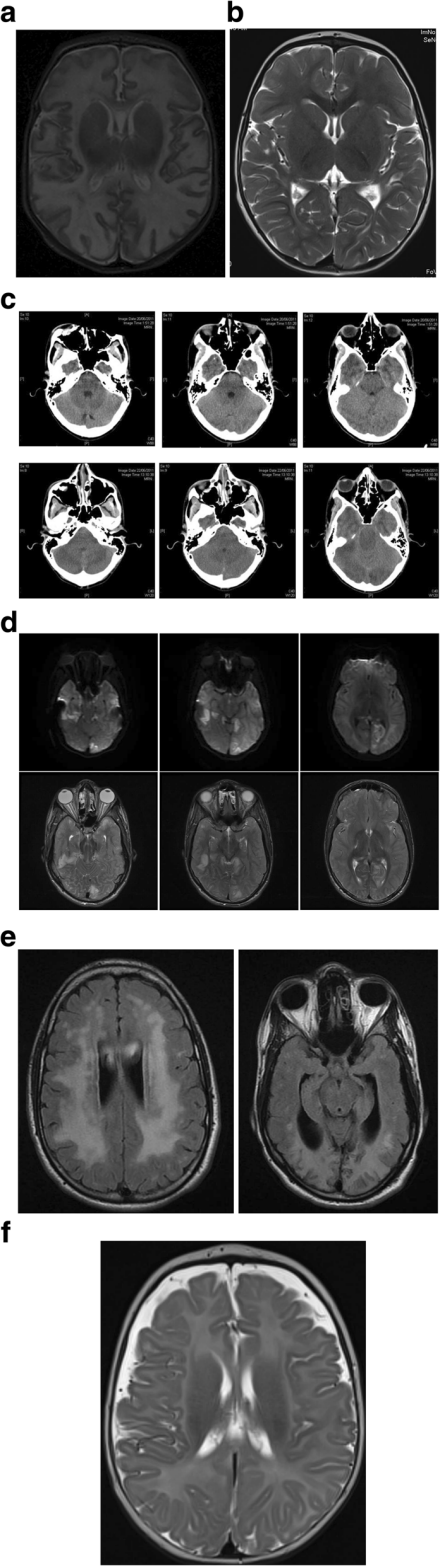
**a**) Sagittal MRI Image of the brain on day 12 of case I with CACTD, indicating extensive abnormal T2 hyperintensity seen within the white matter of both cerebral hemispheres. **b**) T2 -weighted MRI of brain of case 1 at 16 months indicating mild white matter and cortical volume loss especially in the parietal region. Hyperintensity has substantially improved

Case I is a female child of non-consanguineous Caucasian parents, born at term having a hypoglycaemic episode on day two of life. Despite increased feed frequency, she developed hypothermia on day three and was empirically managed as having sepsis with intravenous 10% dextrose and antibiotics. On day 4, after expressed breast milk reintroduction, she became lethargic with an apnoeic episode and seizures. The blood gas demonstrated pH 7∙51 (7∙35–7∙45), pCO2 23 mmHg (32–45) with serum ammonia of 800 μmol/L (10–80). The baby was again placed nil by mouth, restarted 10% dextrose, administered a loading dose of phenobarbitone and was referred to a tertiary metabolic unit. The serum ammonia and blood gas returned to normal in 24 h without any adjunctive ammonia lowering therapy. Initial urine organic acids indicated significant medium and long chain dicarboxylic aciduria without evidence of ketones being produced, indicating a presumptive diagnosis of a long chain MFAO disorder. Initial feeds on day five comprised 90% glucose polymer and 10% expressed breast milk with supplemental S DL-3-OHB of 300 mg/kg/day (Special Products Ltd., UK - (Veriton Pharma)). On day six, the baby deteriorated with multi-organ failure comprising severe cardiac dysfunction with factional shortening 16% (28–45), liver dysfunction and seizures, with her having feeds discontinue, anti-convulsants commence and being invasively ventilated.. However, S DL-3-OHB was continued at a dose of 600 mg/kg/day. Brain MRI demonstrated extensive white matter changes (Fig. 1a). Carbohydrate and MCT based feeds (Polyjoule and Monogen respectively, Nutricia Ltd., USA) restricting long chain dietary fat < 5% of total energy intake were cautiously introduced on day seven per Table 1a. Cardiac fractional shortening had improved to 41% on day nine and she continued to make steady improvement thereafter, being extubated on day nine and discharged home independently feeding on day twenty-three. She had normal early milestones of development and is currently aged 9 years attending normal mainstream school with additional support for attention deficit hyperactivity disorder and mild learning difficulties. There was recovery on brain MRI performed at 2 years of age (Fig. 1b). The diagnosis was subsequently confirmed with skin fibroblast fat oxidation flux studies indicative of either CACTD or CPT2D with leucocyte activity of CPT2 being normal (93% activity of CPT2 relative to controls.) Subsequent molecular studies indicated c.326 + 1 delG mutation and c.50G > C variant of unknown significance of SLC25A20. Contemporary bedside ketone measurements were not performed.

**Table 1:** Energy delivery for each case per day of admission in intensive care, separated as IV (intravenous) and estimated food energy and total for case 1a, CACTD, 1b HMGCL2D and 1c CPT2D. Estimated energy requirement for neonates and infants is 100 Kcal / Kg /day and for adolescent case 1B approximately 50 Kcals / Kg / day

a							
Day	**4**	**5**	**6**	**7**	**8**	**9**	**10**
IV Energy (Kcal)	45	110	95	84	38	16	100
Food energy (Kcal)		11	27	76	172	206	29
Total energy (Kcal)	45	121	122	160	210	222	128
Kcal/Kg	**17**	**47**	**47**	**62**	**81**	**85**	**49**
D,L-3-OHB (mg/kg/day)		300	600	600	600	600	600
b							
Day	**1**	**2**	**3**	**4**	**5**	**6**	**7**
IV Energy (Kcal)	200	510	340	200	0	0	0
Food energy (Kcal)	0	250	1500	2090	1680	2500	2500
Total energy (Kcal)	200	760	1840	2290	1680	2500	2500
Kcal/Kg	**4**	**15**	**37**	**46**	**34**	**50**	**50**
D,L-3-OHB (mg/kg/day)	0	0	0	600	600	600	600
c							
Day	**1 (12 h after admission**	**2**	**3**	**4**	**5**	**6**	**7**
IV Energy (Kcal)	84	76	0	100	0	0	0
Food energy (Kcal)	11	427	517	442	550	551	472
Total energy (Kcal)	95	503	517	542	550	551	472
Kcal/Kg	**21**	**109**	**112**	**118**	**120**	**120**	**103**
D,L-3-OHB (mg/kg/day)	330	978	815	978	978	978	815

### Case 2

**Fig. 2:**
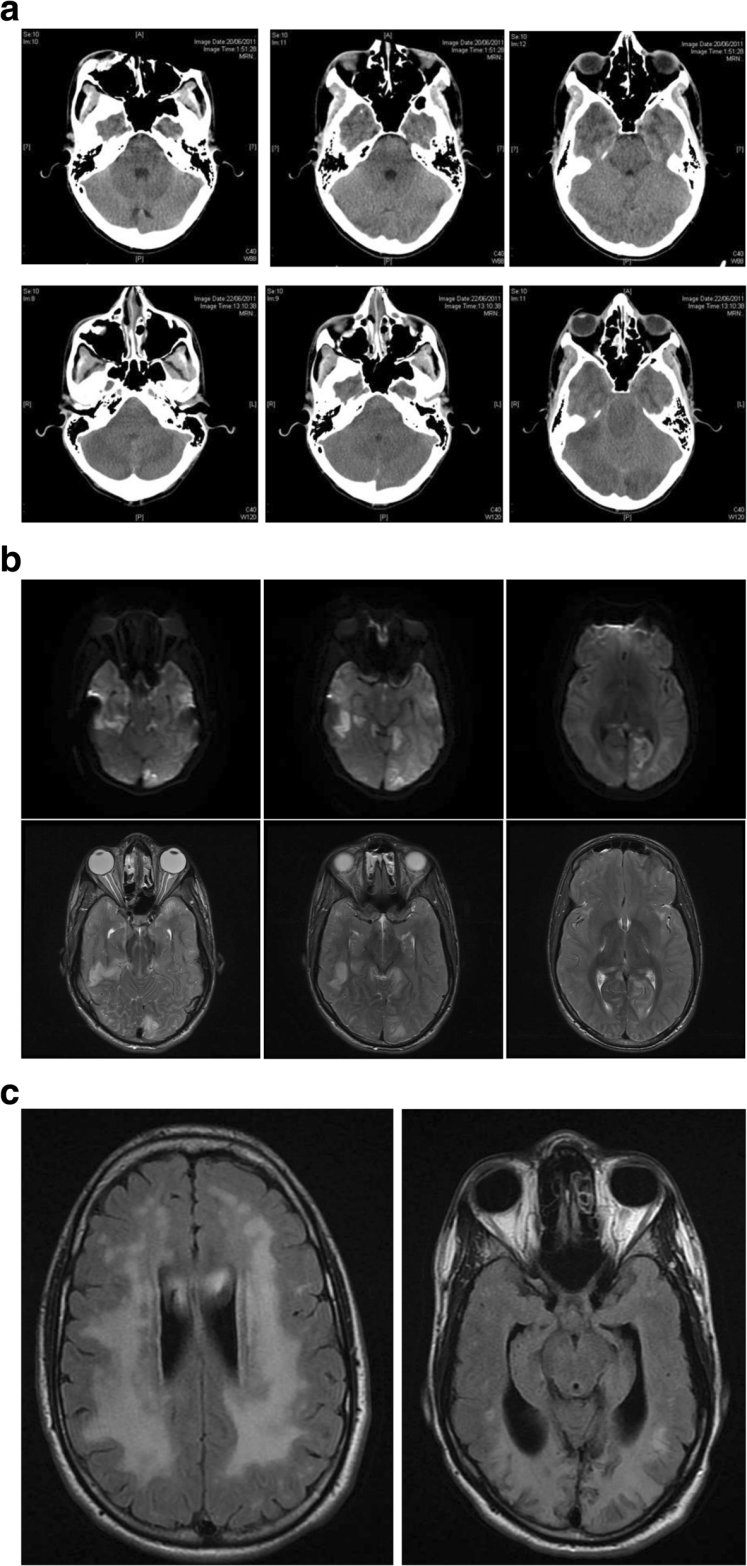
**a**) CT Scan of 16 yr old boy with HMGCL2D on day 1 of admission (top row) when GCS was 10 and on day 3 (bottom row) when GCS was 3, and pupils were dilated bilaterally indicating deteriorating cerebral oedema. **b**) Axial DWI (top row) and T2 (bottom row) images of 16 yr old boy with HMGCL2D on day 3 of admission when GCS was 3. There is infarction involving the occipital and temporal lobes, likely secondary to trans-tentorial herniation and compression of the posterior cerebral arteries. Note also the distended optic nerve sheaths and flattening of the posterior globes, in keeping with significantly elevated intracranial pressure. **c**) Axial T2 Flair images of case 2 with HMGCLD three years after acute life-threatening event (aged 19 years) continuing to demonstrate deep subcortical white matter abnormality with extensive occipital lobe changes leading to cortical blindness

Case 2 was the second male child of non-consanguineous Slovakian parents. He had an uneventful birth and infancy, presenting after four months with hypoglycaemia after switching from breast milk to formula. HMGCL2D was diagnosed on the basis of typical biochemistry including gross elevation of urinary 3-hydroxy-3-methlylglutarate. He was stabilised on a low protein and fat diet but was lost to follow up from age 1 year until the age of 16. At this age he attended normal mainstream education with normal achievement. He presented to the local emergency department after 48 h of persistent vomiting, and was admitted into hospital with lethargy, disorientation and slurred speech. The baseline assessment noted that he had tachycardia, tachypnoea and a core of temperature of 34.7 degrees centigrade. His Glasgow Coma Scale (GCS) was 15. Initial biochemistry demonstrated pH 7.12; Bicarbonate of 6.8 mmol/L (18∙0–24∙0), lactate 16 mmol/L (0–2∙0), Base Excess − 20 (− 2∙0 to + 2∙0), Anion gap 29 mmol/L (8–18) with a blood glucose of 2 mmol/L (3.0–5.5). Acetoacetate level was subsequently reported as 0.07 mmol/L (0.05–0.15) and betahydroxybutyrate 0.01 mmol/L (0.03–3.0). He was resuscitated with dextrose and normal saline boluses, treated with antibiotics and anti-viral agents and maintained on 5% dextrose maintenance fluids. On day two of admission his consciousness level deteriorated with a GCS of 9, being unable to follow commands. He had three beats of clonus on his right side and bilateral extensor plantar responses. Pupils were noted to be reacting sluggishly. A brain CT showed confluent white matter hypodensity and evidence of small ventricles consistent with significantly raised intra-cranial pressure. Serum ammonia was elevated at 455 μmol/L (< 50). Due to compromised neurology, intravenous fluids were restricted to 50 mls/kg/day but were changed to 10% dextrose. Further neurological compromise occurred on day three when he had bilateral fixed and dilated pupils of 7 mm diameter. Repeat CT Brain revealed worsening cerebral oedema (Fig. 2a). A trans-cranial bolt was surgically applied with an opening pressure of 30 mmHg. 600 mg/kg/day of S DL-3-OHB was commenced on day four of hospital admission in tandem with attempts to provide higher energy enteral nutrition as indicated in Table 1b. Aetoacetate measurement increased to 0.11 mmol/L and betahydroxybutyrate to 0.08 mmol/L. Intracranial pressure continued to fluctuate over the subsequent three days but he gradually began to recover, self-ventilating in air by day twenty-four and being discharged home independently ambulatory by day sixty-three. MRI of the brain indicated that he had suffered extensive posterior-circulation infarction, presumed to be due to herniation into the foramen magnum at pressure, and he continues to have complete cortical blindness. Extensive white matter abnormalities were demonstrated at the time of his presentation and persisted three years later on repeat scanning (Fig. 2b and c). The remainder of his neurology remains normal aged 24 years.

### Case 3

**Fig. 3:**
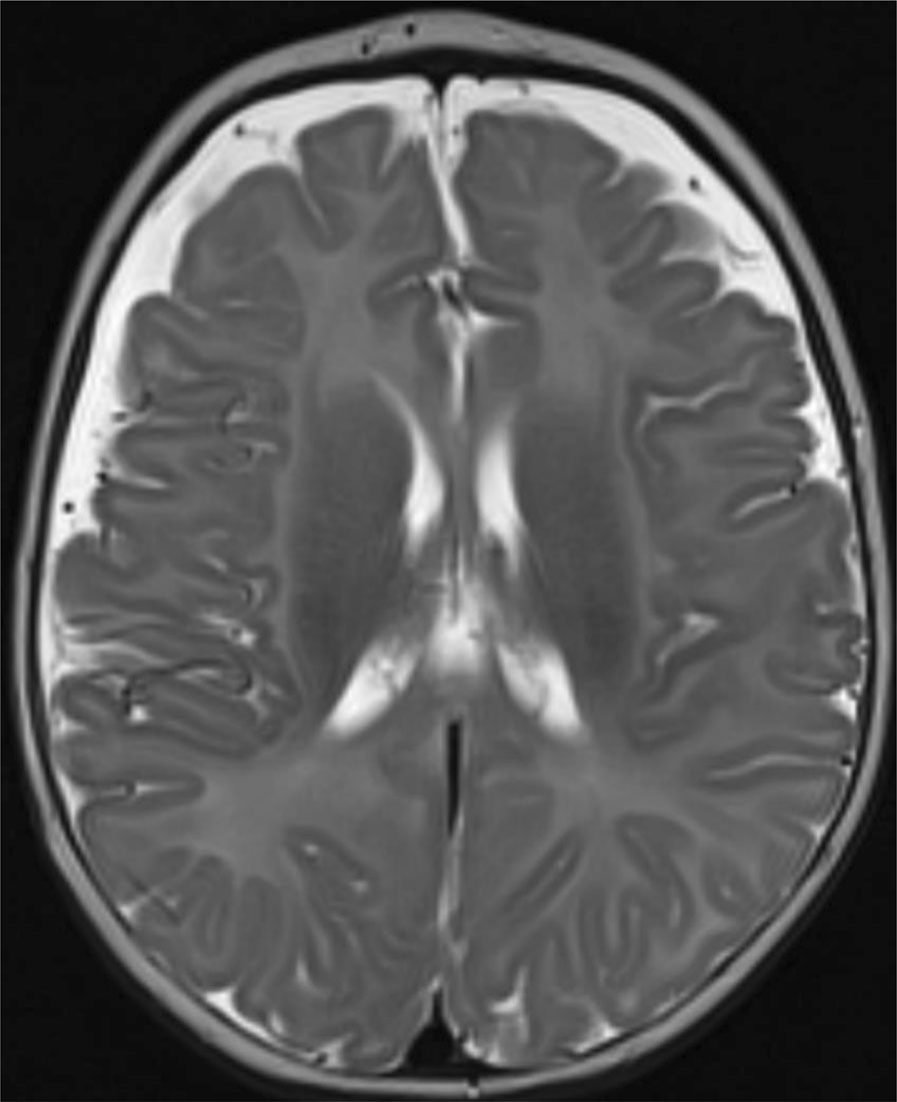
Axial FLAIR image of the brain of case 3 with CPT2D performed at four months of age, one month following her acute decompensation event. Selected image at the level of the lateral ventricles demonstrates prominence of the extra-axial CSF spaces overlying the frontal lobes. Myelin appearance is appropriate for age

Case 3 is the first child of non-consanguineous parents from Syria. She was diagnosed at 3 weeks of age after borderline newborn screening results indicative of CACTD or CPT2D at which time, she was thriving on exclusive breast feeds. Skin fibroblast, white cell enzymology and genetic testing was initiated and subsequently confirmed CPT2D with leucocyte activity being 3% relative to controls. The baby was discharged on a feeding regimen comprising 1/3 of total energy from MCT and 2/3 from breast feeds. At three months of age, over a 48 h period, the baby started refusing MCT based bottle feeds. On the morning of admission, the baby had two vomits over a six hour period and was advised to attend the emergency department (ED). She was assessed in ED to be lethargic but alert with her heart rate of 140 beats per minute (BPM) and core temperature being 37 degrees. Her venous blood gas demonstrated a whole blood glucose level of 1∙5 mmol/L, pH 7∙40, pCO2 27 mmHg, and base excess of − 7∙1 She was treated with two consecutive boluses of 2 ml/kg 10% dextrose and placed on 150% standard maintenance 10% dextrose and normal saline. Two hours after admission she suddenly became flaccid and unresponsive to pain with a core temperature of 32∙6 degrees and heart rate of 100 BPM. Pupils were reactive, the fontanelle not bulging, but there was sustained clonus > 15 beats bilaterally. Venous whole blood glucose was normal at 6∙6 mmol/L with serum ammonia subsequently reported to be 300 μmol/L (10–80).) Bedside beta-hydroxybutyrate measurement was < 0∙2 mmol/L and contemporary measurements of free fatty acid was 3∙96 mmol/L with laboratory beta-hydroxybuyrate of < 0∙18 mmol/L (ratio > 20 implying hypoketosis). Echocardiography performed in the emergency department showed mild septal hypertrophy with no significant cardiac dysfunction. She had clinical evidence of hepatomegaly. After fluid resuscitation and empirical use of intravenous antibiotics, the baby was administered 150 mg/kg/dose of S DL-3-OHB four hourly with the first dose administered less than three hours after the hypothermia event (Table 1c). After twelve hours and three doses of S DL-3-OHB, the baby was awake and beginning to suck with the neurological examination being normal after a further 24 h. Because of the rapid neurological recovery, brain MRI was delayed until one month showing some prominence in frontal extra-axial space, but was otherwise normal (Fig. 3). At one year of age of age, at last review, development was within normal limits being able to pull to stand and standing with support but she does have peripheral hypertonia.. Nonetheless her neurological prognosis remains guarded.

## Discussion

The outcome of severely sick patients with these disorders is dire in the published literature. There are several reports of deaths due to rapidly progressing encephalopathy occurring with decompensation in HMGCLD during adult life, sometimes unexpectedly in previously well individuals [7, 8] [17]. Many cases in earlier series either died or had severe cognitive sequelae. White matter abnormalities are common in this condition although the manifestation does not always correlate with disease course [7, 18]. .Survival in early onset CACT deficiency is rare [4, 19, 20]. Multi-organ failure and death is the usual reported outcome. CP2D is more commonly manifest as an adult form with recurrent rhabdomyolysis, but severe neonatal and infantile forms are well described [21–23]. Sudden death, cardiomyopathy, hypoketotic hypoglycaemia and acute encephalopathy with hyperammonaemia are well described similar to other MFAO disorders.

For all these conditions, patients may enter a vicious cycle of catabolism from which it is often difficult to recover. Catabolism triggers a cascade of issues incorporating accumulation of potentially toxic substrates such as long chain acyl-carnitines and/or failure to generate crucial ketones. For organs in energy deficit, failure to generate ketones compounds critical energy requirements as indicated by these dramatic cases. The third case is interesting in that the encephalopathy occurred after hypoglycaemia was corrected at a time when there was satisfactory glucose provision indicating that an alternate energy source to glucose is required in these instances [24]. This supports the hypothesis that glucose alone is not sufficient to provide cerebral energy requirements in infants [25]. There are data to suggest that both lactate and ketones also play a vital role [26–28].

When patients with MFAO disorders or KB synthetic dysfunction decompensate, the aim is to restore homeostasis, but this is limited by technical difficulties of doing so in critically sick individuals. Often the preferred enteral route may not be effective for macronutrients in the face of persistent vomiting, absorption difficulties and intolerance. As these individuals fluctuate in consciousness, they pose an increasing aspiration risk and hence feeds may be discontinued for protracted periods of time as occurred in these cases. Finally, intravenous therapy has to be restricted in volume as cerebral oedema worsens, as seen dramatically in the second case. Resting energy expenditure studies have demonstrated adult requirements for glucose are approximately 2.5 mg/kg/min [29]. This equates to approximately 2500 ml of 10% dextrose required for an average adult. In a metabolic crisis, significantly more energy will be required. Consequently, fluid restriction compromises energy delivery further. Cytotoxin related cerebral oedema is common in metabolic conditions and thus cerebellar tonsillar herniation is a real risk. Hence it is imperative that treating physicians co-manage optimal energy requirements in conjunction with fluid restrictions, neurological, cardiological and ventilation requirements.

The precedent for the use of S DL-3-OHB has been demonstrated in multiple acyl co-A dehydrogenase deficiency, in which there are several anomalies in mitochondrial ATP production The original paper by van Hove et al. indicated improvement in neurological and cardiac parameters as also seen in our patient with CACT deficiency. Neurological improvements have been reported in other cases using physiological doses of S DL-3OHB, and it has also been successfully used in the glycogen storage disorders to rescue cardiomyopathy [30]. There are experimental data in both animals and humans suggesting that both organs are primed to utilise energy from ketones more effectively in the catabolic state [31, 32]. In the immature brain, ketones can promote myelin production [33]. Whilst cytoplasmic synthesis of mevalonate from HMG Co-A is known to be integral to cholesterol synthesis. There are rat data to suggest that acetoacetate and 3-hydroxybutyrate also may have a significant effect [34, 35]. Hence the MRI findings of significant white matter changes in many cases of HMGLD can be explained by KB deficiency [18]. This hypothesis is yet to be tested fully because the myelin may appear abnormal but does not necessary correlate with function and the composition of this “abnormal” myelin is not clearly understood. In our neonatal CACTD case there is remarkable improvement in myelination across the disease course (Fig. 1b) with therapy in contrast to the HMGCLD case (Fig. 1d).

Stable isotope studies of ketone bodies in neonates indicate that up to 10 kcal/kg of neonatal energy requirements can come from ketones – despite a regular four hourly enteral feeding regimen [24]. The studies estimate that 2 – 3 g/kg/day are transported and likely utilised. These studies are performed in steady state and hence requirements and utilisation could increase in catabolic situations. Racemic S DL-3-OHB comprises D and L stereoisomers of 3-hydroxybutyrate. In terms of energy delivery calculations from S DL–3-OHB, it is difficult to calculate this from the racemic mixture. There have been stable isotope studies in neonates utilising and measuring metabolism of the D-stereoisomer. Similarly clinical studies typically measure the D-isomer even when the racemic mixture has been administered [36]. Studies performed in infant rats indicate that both forms are utilised with a preponderance to utilise the L-stereoisomer for myelin synthesis. Hence, it is difficult to determine from this study the extent to which the different stereoisomers are oxidised or utilised in myelination. In this study pre and post dose ketone measurements were taken before and after treatment for two patients but it is difficult to know when and what samples should be taken as the pharmacokinetics of the S DL-3OHB is not known.

The neonatal brain has relatively high energy requirements, compared to adults and other animals. In a fasting or catabolic state, failure to generate ketones leads to impaired gluconeogenesis which compounds metabolic compromise. At such times, exogenous glucose can rarely be delivered in sufficient quantities to ameliorate energy deficit. Excessive intravenous glucose delivery can perpetuate cerebral oedema and lead to osmotic diuresis leading to further compromise. Providing ketones as a sodium salt can redress this balance whilst also providing an alternative energy substrate that can be utilised by the brain.

Whilst the contemporary use of ketones in this study are suggestive of improvement in clinical conditions, this cannot be proven in this open label uncontrolled study.

Ketogenic diets have become more prevalent in the general community and in specific situations to improve cerebral health such as in pyruvate dehydrogenase complex and GLUT 1 deficiencies as well as generalised epilepsy and Alzheimer’s disease [37–41]. The conditions in this paper are all inherited hypoketotic disorders and hence would deteriorate with a standard ketogenic diet. Nonetheless they demonstrate utility of ketones in acute brain dysfunction which may extrapolate to other situations where there is no IEM.

## Conclusion

This paper demonstrates adjunctive treatment with S DL-3-OHB in severe metabolic decompensation of 3 patients in energy deficit with HMGCLD, CACTD and CPT2D. Their rapidly declining function was recovered with standard treatment and the use of S-DL-3OHB which has led to sustained long-term neurological recovery. This therapy can be considered in instances of impaired ketone production in other critically sick individuals. It is important for investigators utilising such treatment to carefully correlate changes in clinical events with biomarkers to determine efficacy. Ketones may be an adjunctive therapy for other non IEM neurological crises.

## Data Availability

Not applicable.

## Abbreviations

BCA: Bicinchoninic assay
BPM: Beats per minute
CACTD: Carnitine-acyl-carnitine translocase deficiency
CPT2D: Carnitine palmitoyl transferase II deficiency
CT: Computed tomography
ED: Emergency department
FDA: Food and Drugs Administration
GC/MS: Gas chromatography mass spectrometry
GCS: Glasgow Coma Scale
HMGCLD: 3-hydroxyl, 3-methyl, glutaryl CoA lyase deficiency
IEM: Inborn errors of metabolism
KB: Ketone Bodies
MADD: Multiple acyl co A dehydrogenase deficiency
MCT: Medium chain triglyceride
MFAOD: Mitochondrial fatty acid oxidation
MRI: Magnetic resonance image
S DL-3-OHB: Sodium DL-3-hydroxybutyrate
TGA: Therapeutic Goods Administration
UPLC-MS/MS: Ultra-high performance liquid chromatography-tandem mass spectrometry
